# Changes Over Time in the Utilization of Disease-Related Internet Information in Newly Diagnosed Breast Cancer Patients 2007 to 2013

**DOI:** 10.2196/jmir.3289

**Published:** 2014-08-26

**Authors:** Christoph Kowalski, Eva Kahana, Kathrin Kuhr, Lena Ansmann, Holger Pfaff

**Affiliations:** ^1^IMVRUniversity of CologneKoelnGermany; ^2^Department of Sociology and Elderly Care Research CenterCase Western Reserve UniversityCleveland, OHUnited States; ^3^Institute of Medical Statistics, Informatics and EpidemiologyUniversity of CologneKoelnGermany

**Keywords:** disease-related Internet information, digital divide, multilevel analysis, breast neoplasms, hospital differences

## Abstract

**Background:**

As the number of people with Internet access rises, so does the use of the Internet as a potentially valuable source for health information. Insight into patient use of this information and its correlates over time may reveal changes in the digital divide based on patient age and education. Existing research has focused on patient characteristics that predict Internet information use and research on treatment context is rare.

**Objective:**

This study aims to (1) present data on the proportion of newly diagnosed breast cancer patients treated in German breast centers from 2007 to 2013 who used the Internet for information on their disease, (2) look into correlations between Internet utilization and sociodemographic characteristics and if these change over time, and (3) determine if use of Internet information varies with the hospitals in which the patients were initially treated.

**Methods:**

Data about utilization of the Internet for breast cancer–specific health information was obtained in a postal survey of breast cancer patients that is conducted annually in Germany with a steady response rate of 87% of consenting patients. Data from the survey were combined with data obtained by hospital personnel (eg, cancer stage and type of surgery). Data from 27,491 patients from 7 consecutive annual surveys were analyzed for this paper using multilevel regression modeling to account for clustering of patients in specific hospitals.

**Results:**

Breast cancer patients seeking disease-specific information on the Internet increased significantly from 26.96% (853/3164) in 2007 to 37.21% (1485/3991) in 2013. Similar patterns of demographic correlates were found for all 7 cohorts. Older patients (≥70 years) and patients with <10 years of formal education were less likely to use the Internet for information on topics related to their disease. Internet use was significantly higher among privately insured patients and patients living with a partner. Higher cancer stage and a foreign native language were associated with decreased use in the overall model. Type of surgery was not found to be associated with Internet use in the multivariable models. Intraclass correlation coefficients were small (0.00-0.03) suggesting only a small contribution of the hospital to the patients’ decision to use Internet information. There was no clear indication of a decreased digital divide based on age and education.

**Conclusions:**

Use of the Internet for health information is on the rise among breast cancer patients. The strong age- and education-related differences raise the question of how relevant information can be adequately provided to all patients, especially to those with limited education, older age, and living without a partner.

## Introduction

As the number of people with Internet access continues to rise, so too does the number of people using the Internet as a source for health information or health-related activities [1,2]. Over the past two decades, this has led to major changes in both the way health information is consumed and the amount of knowledge laypersons can access relatively easily [3,4].

Breast cancer offers an important arena for exploration of patient Internet use. Breast cancer is a major public health concern, as it is the most common form of cancer and the second major cause of cancer-related deaths among women in the United States. In Germany, 1 in 8 women will face a breast cancer diagnosis in her lifetime [5]. In the Internet age, a new role has become available to patients as information managers. Information acquisition through the Internet can help develop patient competence in dealing with challenges of a life-threatening illness, such as breast cancer [6].

Internet accessibility and its use for health purposes are distributed unequally over the population and its effects are not without controversies. Focusing on benefits to patients, a number of studies emphasize that using health information from the Internet is associated with stronger participation in decision making [7], better decisions [8], more frequent change of health behavior [9], and it may enable patients to communicate with doctors more effectively [10,11]. In contrast, other studies argue that using the Internet for health information may lead to erosion of the patient-provider relationship [12,13] or may confuse patients [14]. The early literature on health-related Internet use was particularly concerned with the limited ability of laypersons to evaluate information obtained on the Internet [15].

It is increasingly important for health care providers to give serious consideration to the information patients collect and to address their understanding of that information [16-18]. Taking into account the varying quality of websites providing health information, quality assurance and expert participation is warranted [19]. Nevertheless, there is indication of improvement in the quality of information offered to patients with breast cancer through a growing number of high quality websites (eg, National Institutes of Health [20], Agency for Healthcare Research and Quality [21], and National Cancer Institute [22] in the United States, and gesundheitsinformation.de [23] and Krebsgesellschaft [24] in Germany). As Eysenbach stated, referring to the accuracy of cancer information websites as far back as 2003: it “is not so bad after all” [10]. The increasing sophistication of Internet sites enables patients to access not only sites designed for patients, but also peer-reviewed scientific articles that describe the latest research relevant to specific problems of the patient.

Patients using the Internet to gain access to health information for various illnesses tend to be younger and of higher socioeconomic status across countries [25-31]. This well-documented “digital divide” might become a major threat to equity in health care once relevant or even necessary information can only be or best be accessed online.

Although reports on the proportion of patients who use the Internet to gain health information vary widely [10,32,33], recent results based on 2011 data suggest that more than 50% of breast cancer patients [25] used the Internet to gain disease-specific information. Because of such widespread reliance on the Internet among female breast cancer patients, there is a clear need for up-to-date information on trends in this form of information acquisition. Differences in the proportion of individuals using the Internet not only differ according to the specific sample and the country or region under investigation, but also study design and the questions posed. Variability among studies in the nature of the disease and time since diagnosis also makes comparisons over time difficult and leaves unanswered questions about trends in the digital divide in relation to health information-seeking [34,35]. Although much research focuses on demographic correlates of online health information use, to our knowledge no study has yet investigated differences across locations of treatment. If variation across locations of treatment persists after controlling for individual characteristics this might offer further important clues to patient motivations for using the Internet for information. Thus, it is possible that unsatisfying experiences in the medical encounter or limited explanations communicated by health care providers would result in increased patient Internet use for health-related information.

The aim of our study was to expand the knowledge base about personal demographic, contextual, and temporal determinants of Internet use among newly diagnosed cancer patients. Specifically, this study aims to (1) present data on the proportion of 7 cohorts of newly diagnosed breast cancer patients treated in German breast center hospitals from 2007 to 2013 who used the Internet for information on their disease, (2) consider stability and change in patient characteristics predicting Internet use over time focusing on the digital divide based on age, education, and insurance status as an indicator of socioeconomic status, and (3) determine if use of information from the Internet varies by the hospital in which the patients were initially treated.

In doing so, we hope to expand existing knowledge by investigating developments over time and addressing the health care organization’s contribution to online health information use while taking clinical data (stage, type of surgery) and potentially relevant patient characteristics (partnership status, native language, gender) into account.

## Methods

### Participants

This report analyzed data drawn from a larger program of research designed to investigate the breast center concept of the German federal state of North Rhine-Westphalia (population 17.5 million). Patients treated for newly diagnosed breast cancer in one of the accredited breast center hospitals were asked to self-administer a questionnaire at home after discharge from the hospital [36]. Patients were included in the survey if they had a first diagnosis of breast cancer, underwent surgery during their current hospital stay, and had at least one malignancy, at least one postoperative histology, and a confirmed diagnosis of breast cancer with an International Classification of Diseases (ICD) code of C50.x or D05.x. Each year between February and June (survey period 6 months), all patients who fulfilled the inclusion criteria were included in the study consecutively. Cross-sectional surveys were performed with samples of patients from all accredited breast center hospitals in the region studied.

Shortly before discharge from the hospital, patients were asked by the hospital staff to give written consent to be included in the survey. Once the patients had given their consent, hospital personnel from the centers provided the research team with clinical information on the patients. The survey was designed according to Dillman’s Total Design Method with 3 contacts [37]. The survey was sent out to the patient’s home address within a week of receiving written consent. The study was approved by the institutional ethics committee of the University Hospital of Cologne, Germany. We analyzed data from each of the 7 years (2007 to 2013). Of the 35,371 patients meeting the inclusion criteria, 31,293 (88.47%) consented to the survey. Of these, 27,491 (87.85%) returned the questionnaire. These patients make up the sample for the analyses.

### Measures

#### Dependent Variable

The dependent variable was use of the Internet for breast cancer–specific health information assessed based on response to a survey question that asked about such Internet use (yes/no).

#### Independent Variables

Patient sociodemographic data and clinical status served as independent variables. Patients were asked to indicate their date of birth, native language, insurance status, highest year of education attained, and partnership status on the questionnaire. Except for age (continuous) the sociodemographic variables are categorized into native language (German vs other), insurance status (statutory health insurance vs partly private/partly private), highest year of education (≥10 years of school vs <10 years of school), partnership status (living with a partner vs not living with a partner), and gender (male vs female).

In addition to the data collected by the patient questionnaire, medical personnel contributed clinical data and information about type of surgery performed after patient consent. The cancer stage was categorized using Union for International Cancer Control (UICC) categories [38]; type of surgery was dichotomized (breast-conserving treatment vs mastectomy).

### Statistical Analyses

#### Proportions of Internet Users

The proportion of patients who used Internet information about breast cancer was calculated separately for each of the 7 cohorts, both overall and stratified for younger patients with more formal education to spotlight the digital divide (age <50 years; ≥10 years of school) and older patients with less formal education (age ≥70 years; <10 years of school). To test for differences over time, the Cochran-Armitage trend test was applied. In addition, the share of the 4 groups that resulted when stratifying for age and education among Internet users was analyzed. We performed bivariate tests to examine associations between the independent variables included in the model. We conducted chi-square tests for associations between all categorical variables (type of surgery, native language, years of schooling, insurance status, living with a partner, gender, cancer stage). Spearman rank correlation was used to examine the correlation between age and the ordinal variable cancer stage. Also, *t* tests were conducted to examine age differences for the different groups in the dichotomous variables. The cross-year dataset was used for these analyses.

#### Multilevel Models

Data from each survey cohort were analyzed separately and in an overall model using multilevel analysis. This is the method of choice when accounting for the nested structure of the data, such as patients (level 1) in hospitals (level 2) [39]. Two-level models without predictors were fitted to yield the intraclass correlation coefficient (ICC) for the null model. The ICC represents the proportion of the variance of the dependent variable attributable to the hospital level. In a second step, all patient characteristics were included. A number of patients indicated they did not have access to the Internet in an earlier question and did not respond to the dependent variable. To avoid case deletion, cases that indicated they did not have access to the Internet in the earlier question were coded as not having used the Internet. Cases with missing data in the dependent variable and missing data in this earlier question were excluded from all analyses (n=1022). Patients with missing data in the continuous age variable were excluded in the multilevel models (n=243), leaving 26,226 patients for the multilevel analyses. Missing data on all other independent variables were included in the model as separate categories to avoid case deletion, and omitted in the results tables. The ICCs of these models represent the proportion of variance attributable to the hospital-level characteristics after accounting for variation in the patient characteristics, (ie, the different patient case mix). Because of the small ICCs, no hospital-level characteristics were included in the models. The overall model included a cohort variable to account for the survey year. In addition, we included a gender variable that we did not include in the year-by-year analyses because of small strata. SPSS version 22.0 (IBM Corp, Armonk, NY, USA) was used for descriptive analysis and MLWiN 2.25 (Centre for Multilevel Modelling, Bristol, UK) for multilevel analysis. R 3.0.2 (R Project for Statistical Computing, Vienna, Austria) was used to calculate the Cochran-Armitage trend test.

## Results

Table 1 shows the percentage of breast cancer patients who reported they used the Internet to obtain information about their disease. There was a relatively steady, statistically significant increase in this percentage over the 7-year study period (2007: 26.96%, 853/3164; 2013: 37.21%, 1485/3991; χ^2^_1_=138.0, *P*<.001). No relevant changes were found for the proportion of younger, higher-educated patients who used the Internet (χ^2^_1_=0.4, *P*=.51). Proportions for this group remained relatively stable, between 60% and 70% throughout the study period. The proportion of older patients with little formal education who used Internet information increased significantly from 2007 (2.9%, 13/444) to 2013 (4.7%, 29/617; χ^2^_1_=6.8, *P*=.009) but remained below 6% for all cohorts. Among men, the overall proportion was only 25.4% (32/126, not presented in a table).

**Table 1:** Patients reporting to have used the Internet to obtain information about breast cancer across the entire study period (2007-2013) and by younger, higher-educated patients and older, less-educated patients.

Year	Overall		Age <50 years; ≥10 years of school		Age ≥70 years; <10 years of school	
	n/N	%	n/N	%	n/N	%
2007	853/3164	26.96	283/452	62.6	13/444	2.9
2008	1093/3689	29.63	331/547	60.5	14/517	2.7
2009	1196/3855	31.02	355/559	63.5	20/614	3.3
2010	1272/3767	33.77	407/606	67.2	30/576	5.2
2011	1343/3940	34.09	397/635	62.5	26/668	3.9
2012	1505/4063	37.04	413/634	65.1	37/666	5.6
2013	1485/3991	37.21	401/642	62.5	29/617	4.7

To better understand which patient group contributed most to the increase in Internet use, we compared the share in users for 4 different groups: (1) age ≥70 years, <10 years of school; (2) age <50 years, <10 years of school; (3) age ≥70 years, ≥10 years of school; and (4) age <50 years, ≥10 years of school (Table 2). None of the 4 groups’ share of Internet users increased substantially over time.

**Table 2:** Composition of Internet health information users.

Patient subgroup^a^	Year, n/N^b^ (%)						
	2007	2008	2009	2010	2011	2012	2013
Age ≥70 years; <10 years of school	13/824 (1.6)	14/1074 (1.30)	20/1181 (1.69)	30/1268 (2.37)	26/1333 (1.95)	37/1498 (2.47)	29/1464 (1.98)
Age <50 years; <10 years of school	54/824 (6.6)	74/1074 (6.89)	74/1181 (6.27)	75/1268 (5.91)	72/1333 (5.40)	63/1498 (4.21)	63/1464 (4.30)
Age ≥70 years; ≥10 years of school	10/824 (1.2)	20/1074 (1.86)	20/1181 (1.69)	26/1268 (2.05)	41/1333 (3.08)	60/1498 (4.01)	68/1464 (4.64)
Age <50 years; ≥10 years of school	283/824 (34.3)	331/1074 (30.82)	355/1181 (30.06)	407/1268 (32.10)	397/1333 (29.78)	413/1498 (27.57)	401/1464 (27.39)

^a^Patients aged 50 to 69 years comprise the remaining portion of the sample.

^b^The N’s presented represent only the patients with valid data for education and age.

Tables 3 to 5 present bivariate associations between the independent variables in the sample. Most notably, partnership and insurance status were significantly correlated with many other study variables, such as type of surgery, native language, stage, and education. Age differences were found for type of surgery, native language, years of schooling, partnership status, and gender. Spearman rho was .069 (*P*<.001) for the correlation between stage (ordinal) and age (not presented in a table).

**Table 3:** Bivariate associations between independent variables in the sample: dichotomous variables, percentages, and Pearson chi-square^a^ (N=27,491).

Variable		Mastectomy, %				Native language, %				Years of school, %				Private health insurance,^c^ %				Living with partner, %			
		Yes	No^b^	χ^2^_1_	*P*	German	Other	χ^2^_1_	*P*	<10	≥10	χ^2^_1_	*P*	Yes	No	χ^2^_1_	*P*	No	Yes	χ^2^_1_	*P*
**Gender**																					
	Female	26.6	73.4	285.9	<.001	94.6	5.4	3.5	0.08	51.6	48.4	0.7	0.43	24.6	75.4	2.0	0.12	28.1	71.9	7.6	.006
	Male	93.7	6.3			90.8	9.2			55.4	44.6			29.9	70.1			17.3	82.7		
**Mastectomy**																					
	Yes					95.0	5.0		0.09	52.9	47.1	5.2	0.02	23.2	76.8	10.8	0.001	33.9	66.1		<.001
	No^b^					94.5	5.5	3.0		51.3	48.7			25.2	74.8			26.0	74.0	155.4	
**Native language**																					
	German									52.4	47.6	121.8	<.001	25.5	74.5	179.8	<.001	28.3	71.7	26.0	<.001
	Other									37.1	62.9			9.8	90.2			22.2	77.8		
**Years of school**																					
	<10													13.7	86.3	1883.8	<.001	29.4	70.6	33.4	<.001
	≥10													37.1	62.9			26.2	73.8		
**Private health insurance** ^c^																					
	Yes																	24.4	75.6	58.7	<.001
	No																	29.4	70.6		

^a^Pairwise deletion used in the chi-square analysis, Fisher’s exact test.

^b^Breast-conserving treatment.

^c^Yes: (partly) private health insurance; no: only statutory health insurance.

**Table 4:** Bivariate associations between cancer stage and dichotomous independent variables in the sample: percentages and Pearson chi-square^a^ (N=27,491).

Variable		Cancer stage, n/N (%)					χ^2^_4_	*P*
		Stage 0	Stage 1	Stage 2	Stage 3	Stage 4		
**Mastectomy**							3078.1	<.001
	Yes	426/1484 (28.71)	1264/9953 (12.70)	2394/8029 (29.82)	1541/2505 (61.52)	496/806 (61.54)		
	No^b^	1058/1484 (71.29)	8689/9953 (87.30)	5635/8029 (70.18)	964/2505 (38.48)	310/806 (38.46)		
**Native language**							7.2	
	German	1405/1480 (94.93)	9434/9930 (95.01)	7624/8096 (94.17)	2386/2529 (94.35)	798/839 (95.11)		
	Other	75/1480 (5.07)	496/9930 (4.99)	472/8096 (5.83)	143/2529 (5.65)	41/839 (4.89)		
**Years of school**							53.9	<.001
	<10	701/1473 (47.59)	5001/9878 (50.63)	4145/7936 (52.23)	1386/2480 (55.89)	493/825 (59.76)		
	≥10	772/1473 (52.41)	4877/9878 (49.37)	3791/7936 (47.77)	1094/2480 (44.11)	332/825 (40.24)		
**Private health insurance** ^c^							55.0	<.001
	Yes	390/1458 (26.75)	2570/9712 (26.46)	1828/7912 (23.10)	545/2481 (21.97)	158/834 (18.94)		
	No	1068/1458 (73.25)	7142/9712 (73.54)	6084/7912 (76.90)	1936/2481 (78.03)	676/834 (81.06)		
**Living with partner**							88.4	<.001
	No	380/1478 (25.71)	2515/9947 (25.28)	2374/8062 (29.45)	837/2515 (33.28)	261/830 (31.45)		
	Yes	1098/1478 (74.29)	7432/9947 (74.72)	5688/8062 (70.55)	1678/2515 (66.72)	569/830 (68.55)		
**Gender**							32.9	<.001
	Male	4/1510 (0.26)	31/10,127 (0.31)	43/8252 (0.52)	27/2580 (1.05)	10/861 (1.2)		
	Female	1506/1510 (99.74)	10,096/10,127 (99.69)	8209/8252 (99.48)	2553/2580 (98.95)	851/861 (98.8)		

^a^Pairwise deletion used in the chi-square analysis.

^b^Breast-conserving treatment.

^c^Yes: (partly) private health insurance; no: only statutory health insurance.

**Table 5:** Bivariate associations between age and dichotomous independent variables in the sample (N=27,491).

Variable		Mean age	*t* (*df*)	*P*
**Mastectomy**				<.001
	Yes	62.5	–14.96 (10,537.5)	
	No^a^	59.7		
**Native language**				<.001
	German	60.7	–20.88 (1666.8)	
	Other	54.6		
**Years of school**				<.001
	<10	64.5	–63.59 (25,574.3)	
	≥10	55.8		
**Private health insurance** ^b^				.587
	Yes	60.5	–0.54 (11,474.2)	
	No	60.4		
**Living with partner**				<.001
	No	64.6	–35.83 (11,961.4)	
	Yes	58.6		
**Gender**				<.001
	Male	66.2	5.62 (27,216)	
	Female	60.4		

^a^Breast-conserving treatment.

^b^Yes: (partly) private health insurance; no: only statutory health insurance.

**Figure 1:**
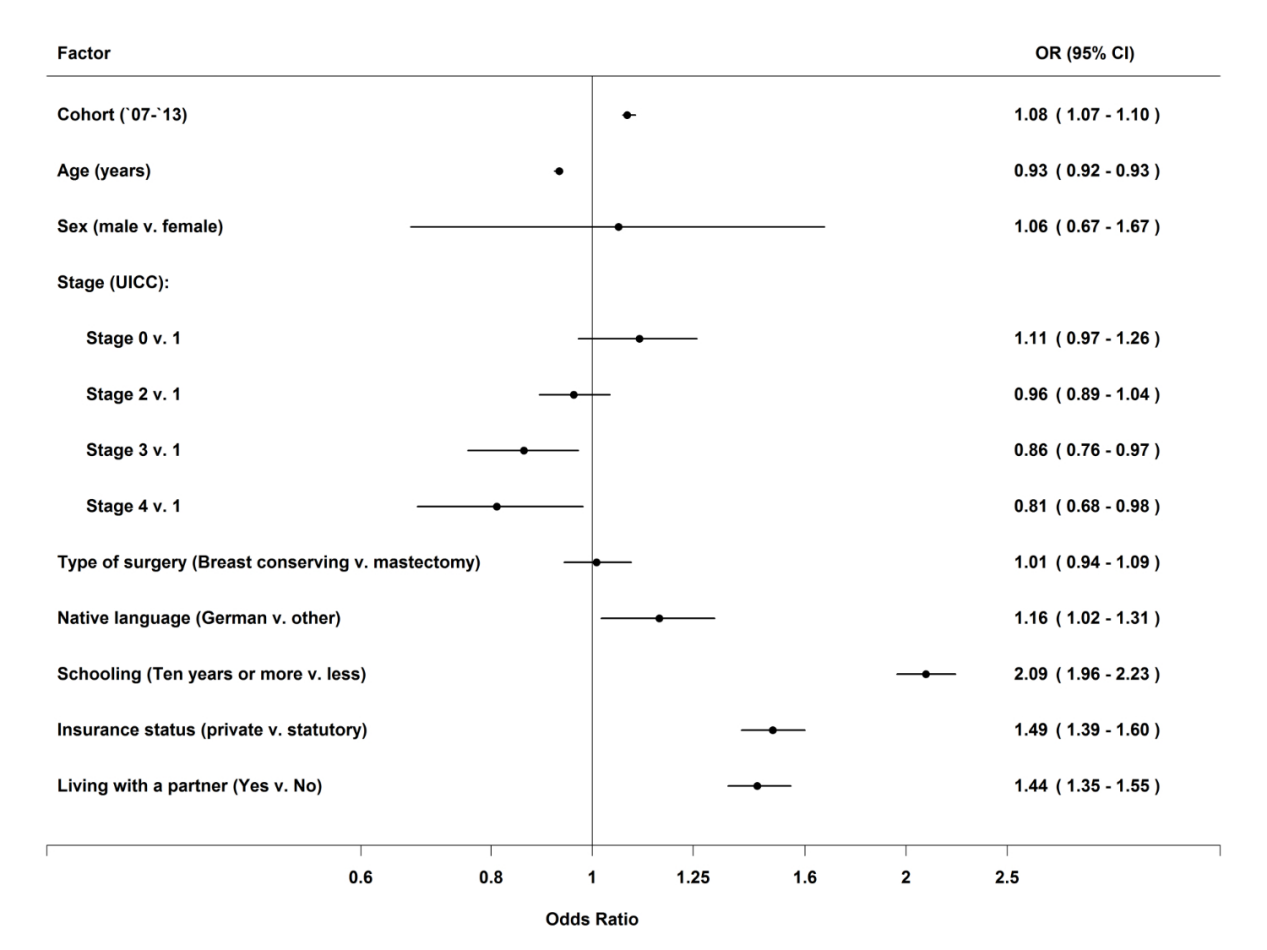
Logistic multilevel regression analysis on having used the Internet to obtain information about the disease for the overall model cohorts from 2007-2013 (N=26,226).

Results from the multilevel analyses are presented in Table 6 and Figure 1. Table 6 presents results for each single cohort and Table 1 for the overall model (ie, an across-years analysis that includes an additional year variable). Table 6 and Figure 1 reveal associations between health-related Internet use and age and education, with higher formal education (OR 2.09, 95% CI 1.96-2.23) and decreasing age (OR 0.93, 95% CI 0.92-0.93) being significantly associated with higher Internet information use in the overall model. In addition, patients who were privately or partly privately insured (OR 1.49, 95% CI 1.39-1.60) or were living with a partner (OR 1.44, 95% CI 1.35-1.55) were more likely to use the Internet for breast cancer–related information. Each of the cohorts yielded the same statistically significant predictors of Internet use (except for stage in 2011 and partner in 2013) with only slight differences in effect sizes. A foreign native language vs German (OR 1.16, 95% CI 1.02-1.31) and cancer stages 3 (OR 0.86, 95% CI 0.76-0.97) and 4 (OR 0.81, 95% CI 0.68-0.98) vs cancer stage 1 were found to be statistically significantly associated with less Internet use in the overall model only. Type of surgery (OR 1.01, 95% CI 0.94-1.09) and gender (OR 1.06, 95% CI 0.67-1.67) were not associated with Internet use. ICCs of the dependent variable in the null models were small (≤0.03) for all 7 cohorts, especially after introducing patient level predictors, indicating small differences between hospitals (2007: 0.03 after including patient characteristics, 0.03 for the null model; 2008: 0.01, 0.03; 2009: 0.01, 0.02; 2010: 0.00, 0.01; 2011: 0.00, 0.01; 2012: 0.02, 0.03; 2013: 0.01, 0.03).

**Table 6:** Logistic multilevel regression analyses on having used the Internet to obtain information about the breast cancer (N=26,226).

Variable		Year, OR (95% CI)						
		2007 n=3078	2008 n=3630	2009 n=3816	2010 n=3760	2011 n=3920	2012 n=4053	2013 n=3969
**Constant**								
		15.27 (7.20-32.41)	23.82 (11.98-47.35)	24.00 (12.35-46.68)	15.37 (8.15-28.98)	11.62 (6.33-21.35)	13.70 (7.56-24.83)	21.04 (11.67-37.96)
**Age (years)**								
		0.92 (0.91-0.93)	0.92 (0.91-0.93)	0.93 (0.92-0.93)	0.93 (0.92-0.94)	0.93 (0.93-0.94)	0.94 (0.93-0.94)	0.93 (0.93-0.94)
**Stage (ref: stage 1)**								
	Stage 0	0.94 (0.61-1.43)	1.04 (0.74-1.45)	1.37 (0.97-1.94)	0.95 (0.64-1.41)	1.36 (0.97-1.92)	1.06 (0.78-1.45)	1.10 (0.81-1.51)
	Stage 2	1.15 (0.91-1.44)	0.94 (0.77-1.15)	0.95 (0.78-1.16)	1.07 (0.88-1.30)	0.94 (0.78-1.13)	0.86 (0.71-1.03)	0.95 (0.79-1.44)
	Stage 3	0.90 (0.63-1.27)	0.78 (0.57-1.07)	0.81 (0.59-1.12)	0.82 (0.60-1.12)	0.97 (0.72-1.31)	0.85 (0.63-1.14)	0.80 (0.59-1.08)
	Stage 4	1.60 (0.89-2.86)	0.64 (0.37-1.13)	1.19 (0.72-1.97)	1.02 (0.66-1.58)	0.60 (0.37-0.97)	0.67 (0.39-1.13)	0.73 (0.46-1.15)
**Type of surgery**								
	Breast conserving (vs mastectomy)	0.90 (0.72-1.12)	1.20 (0.97-1.48)	0.96 (0.79-1.17)	1.05 (0.86-1.28)	0.93 (0.77-1.13)	0.98 (0.82-1.18)	1.00 (0.83-1.21)
**Native language**								
	German (vs other)	1.32 (0.87-2.02)	0.96 (0.67-1.38)	1.02 (0.71-1.45)	1.30 (0.93-1.81)	1.34 (0.98-1.83)	1.02 (0.76-1.37)	0.91 (0.69-1.21)
**Years of schooling**								
	≥10 (vs <10)	2.40 (1.96-2.95)	1.92 (1.60-2.30)	2.13 (1.79-2.53)	1.84 (1.55-2.17)	1.99 (1.68-2.35)	2.02 (1.71-2.38)	2.20 (1.86-2.59)
**Insurance status**								
	(Partly) private (vs statutory)	1.31 (1.06-1.62)	1.83 (1.50-2.22)	1.31 (1.07-1.58)	1.84 (1.53-2.21)	1.37 (1.14-1.65)	1.28 (1.07-1.53)	1.48 (1.25-1.76)
**Living with a partner**								
	Yes (vs no)	1.50 (1.19-1.88)	1.44 (1.18-1.76)	1.38 (1.14-1.68)	1.31 (1.09-1.57)	1.47 (1.23-1.77)	1.52 (1.27-1.81)	1.13 (0.96-1.34)

## Discussion

Findings of this study expand the discussion about the role of computer technology to facilitate proactive illness management for breast cancer patients. Patients diagnosed with breast cancer have creatively used the Web to meet the challenges of their illness. It has been argued that these patients face substantial barriers as they try to make sense of their illness in a fragmented and limited information environment [40].

Our findings confirm expectations that the use of the Internet for seeking health information is on the rise among breast cancer patients. As hypothesized, being younger and having a higher level of education increased the likelihood that a patient would search the Internet for information about their disease. Two-thirds of patients younger than 50 years and with more than 10 years of schooling used the Internet, whereas less than 6% of those older than 70 years and with less than 10 years of schooling did so in all 7 cohorts, with only small changes over time. In addition, living with a partner and having private insurance was positively associated with Internet information use. Only small variation in the dependent variable was found between hospitals and over time. This indicates that there is no systematic impact of the treating institution on the patients’ decision to use Internet information. In aggregate, these data indicate that personal demographic factors play a much greater role in shaping proactive involvement in searching for health information than do situational aspects of the health care environment.

We were able to show a small association for cancer stage and Internet use only in the overall model, pointing to a more limited role of illness characteristics among personal determinants of Internet use [30]. Studies with population-based samples often found that individuals who reported impaired health or chronic conditions used the Internet more frequently (eg, [41,42]). Our sample consisted of individuals who suffered from an acute and life-threatening disease; therefore, they were relatively homogenous with regard to the health status.

A substantial digital divide was found in our study with respect to age and formal education and it did not clearly decrease over time. This finding is inconsistent with suggestions in the literature that the digital divide may be disappearing [43]. Given that partnership status of the patients in our study contributed to Internet use, it is apparent that some patient groups are systematically excluded from one of the most common contemporary sources of information. This raises concerns about alternative methods for meeting pressing information needs of patients encountering a diagnosis that poses great uncertainty. Furthermore, these patients have little access to interventions and practical tools involving computers or advanced electronic devices, such as smartphones or tablet computers [44]. Discovering how these patient groups can be adequately approached is a central task for future health communication efforts.

Privately insured patients used the Internet to search for information on breast cancer significantly more often than their counterparts. This finding is consistent with prior research [45] and is most likely due to the higher socioeconomic status of privately insured patients. This is not fully captured by the education variable in the models. A population-based survey from the United States showed that people living in rural areas used the Internet less than their urban counterparts did both in general and for health-specific purposes. This difference is mainly due to the differences in socioeconomic status and accessibility of broadband [46]. This not only jeopardizes aims of equal access to health information, but might also lead to worse quality of care and a confounding factor when comparing providers. Our data do not suggest that such an effect exists in our specific sample, since differences between hospitals (which controls for rural/urban differences to some extent) were small.

Patients who were not native speakers of German were found to use the Internet less often only in the overall model. However, it must be considered that patients with difficulties understanding German are likely to be underrepresented in this sample because the questionnaire was administered in German. This is relevant to interpreting bivariate associations with the native language variable. However, statistically adjusting for this would require more knowledge about nonrespondents with a native language other than German. Research has shown that the degree to which a person is comfortable speaking a language other than his or her own affects both use of and trust in health information sources [47]. This does not contradict the previously tested diversification hypothesis in which minority status, not native language, proven to shape health-relevant Internet use [48]. The finding of lesser Internet use in nonnative speakers as well as the strong decrease of Internet information use with decreasing formal education also reflects inaccessibility of Internet based health information to patients with low health literacy. Much of the information that is accessible online exceeds the reading level that is recommended for general use by information-seeking patients [49,50].

It has been reported that the prevalence of seeking health information on the Internet is higher in women compared to men [51]. In our sample, we found no gender differences when taking other patient characteristics into account. Because male breast cancer patients tend to have difficulties accessing important information through traditional channels [52,53], it is somewhat surprising that they do not use the Internet more than female patients.

Online information can be a central resource for the elderly who may have difficulty in accessing health information because they are homebound and/or have little social support [54]. This requires a careful investigation of what might help increase the number of older adult Internet users. Some attention has been given to factors that contribute to the lower rates of Internet information use among the older population besides physical impairment, less access, and less familiarity. For example, older adults may distrust the information provided online [55]. Select, expert-guided, quality assured information that is recommended by health care providers might be a key to reaching this skeptical patient subgroup. Additionally, older adults may benefit from training and from availability of more senior-friendly design. This strategy has long been advocated [56].

The third group that needs to be focused on is the part of the population with low formal education and limited health literacy to avoid the reinforcement of existing social differences [57,58]. The more information that is available online, the more important it is to also provide them through other sources of communication for those who do not have access to the Internet or are not Internet savvy.

A number of limitations of our study need to be mentioned. The study sample is a highly specific subsample of the general population and this limits generalizability of findings. Another limitation is the general nature of the Internet usage measure that does not specify types of information that was actually accessed and how this relates to patient preferences [59]. As each cohort was surveyed only once in our study, we cannot establish whether seeking Internet information is a result of the experience within the hospital or independent of it. Further research is required to investigate the temporal order [18,60]. However, the small ICCs hint at little impact of the institutional context. Also, comparing proportions of patients using the Internet to obtain disease-specific information with results from other studies should be done with caution. Patients in this study responded to the survey shortly after discharge from the hospital and it is possible that some patients consulted the Internet later on. We were able to detect significant associations between Internet use and native language as well as cancer stage only in the overall model with the higher statistical power as compared to the year-by-year analyses.

Despite its limitations, the present study is ground breaking in providing a detailed description of Internet information use in 7 consecutive cohorts of seriously ill patients from a specific set of hospitals that allows for the analysis of change over time. This is also a first effort to consider whether Internet use is linked to differences between the health care organizations.

Women diagnosed with breast cancer have wide-ranging information needs. In a study of Internet savvy younger women (younger than 45 years) diagnosed with breast cancer, results suggested that these patients searched for information to help them make good treatment decisions, to learn about their future care and prospects, and to pursue social support [61]. These goals are congruent with principles of patient empowerment and involvement in health care decision making.

This study also has implications for practice that has not yet fully harnessed the healing and empowerment potential of technology for the benefit of persons living with life-threatening illnesses. Access to the information on the Internet has been shown to enhance health-promoting behaviors [62,63].

## Abbreviations

ICC: intraclass correlation coefficient
ICD: International Classification of Diseases
UICC: Union for International Cancer Control
